# Benign juvenile idiopathic epilepsy in captive Iberian lynx (*Lynx pardinus*) in the ex situ conservation program (2005–2019)

**DOI:** 10.1186/s12917-021-02868-z

**Published:** 2021-04-15

**Authors:** Juan J. Mínguez, Yasmin El Bouyafrouri, José A. Godoy, Antonio Rivas, Jesús Fernández, Victoria Asensio, Rodrigo Serra, María J. Perez-Aspa, Valentina Lorenzo

**Affiliations:** 1 Hospital Veterinario Guadiamar, Sanlúcar la Mayor, Seville, Spain; 2 Pride Veterinary Centre, Derby, UK; 3 Centro de cría del lince ibérico El Acebuche-OAPN/Tragsatec. Parque Nacional de Doñana, Huelva, Spain; 4 Departamento de Ecología Integrativa, Estación Biológica de Doñana (EBD-CSIC), Sevilla, Spain; 5 Departamento de Mejora Genética Animal, INIA, Madrid, Spain; 6 Centro de Cría en cautividad de Lince ibérico (CCLI) de Zarza de Granadilla, Cáceres, Spain; 7 Centro Nacional de Reprodução de Lince Ibérico, Silves, Portugal; 8 Centro de Cría en cautividad de Lince ibérico (CCLI) la Olivilla. Agencia de Medio Ambiente y Agua de Andalucia, Jaen, Spain; 9 NeurologiaVeterinaria, Getafe, Madrid Spain

**Keywords:** Feline, Wild cat, Lynx, Seizure, Idiopathic epilepsy, Juvenile epilepsy, Benign epilepsy

## Abstract

**Background:**

Benign juvenile idiopathic epilepsy has been described in humans but rarely in animals. The objectives of the study were to describe the clinical signs, clinical data, imaging findings, genetic examinations, treatment, long-term outcome and prognosis in Iberian lynx with juvenile epilepsy. Medical records, video recordings and diagnostic data from 2005 to 2019 were reviewed.

**Results:**

Twenty lynx cubs with early onset of epileptic seizures (ES) from the conservation program were included. The average age at seizure onset was 75 days. Isolated and cluster ES were recorded. Focal ES, focal ES evolving into generalized ES with a stereotypical pattern and generalized ES were observed. All the cubs were normal between episodes, had a normal neurological examination and unremarkable investigations. Phenobarbital was used as a first line antiepileptic drug (AED). ES halted 10 days (0–34) after starting treatment in eight out of twenty cubs (40%). Treatment was discontinued in this group after a mean of 578 days and no further ES were reported (mean follow-up longer than 5 years). Eleven animals (55%) continued on AED treatment for a mean of 1306 days (70–3466). An adult-onset was observed for one lynx (5%). Polytherapy was necessary in seven lynxes (35%). The inheritance pattern observed was compatible with an autosomal recessive condition. Based on this assumption, mating between two identified carriers has been avoided since 2012, which may have contributed to the subsequent decrease in prevalence, with no further cases detected in 2018 and 2019.

**Conclusions:**

*Lynx pardinus* may have an early onset self-limiting ES syndrome characteristic of benign juvenile idiopathic epilepsy. Information obtained from this study strongly suggests a genetic basis for the here presented epilepsy.

## Background

Epileptic seizures (ES) have been rarely reported in the Iberian Lynx [1, 2]. Idiopathic epilepsy is defined as a disease of the brain characterized by an enduring predisposition to generate seizures with no underlying disease, in which a causative genetic basis has been suspected, identified or confirmed [3].

Benign juvenile idiopathic epilepsy has been described in humans but rarely in animals, where it has been only reported in the Lagotto Romagnolo dog, Arabian foals and recently, in one Iberian Lynx cub. These group of epilepsies are characterized by early onset with no underlying aetiology, rapid control of ES, and self-limiting course [4–7]. This disorder may have an impact in the management of breeding programs for endangered species, such as the Iberian Lynx [8, 9]. The aims of this study are to report the occurrence and clinical data of a juvenile benign form of epilepsy in a population of Iberian Lynx, to classify the lynx epilepsy and to assess its potential genetic basis.

## Results

### Clinical findings

20 out of the 403 lynx cubs from the conservation program were affected by benign juvenile idiopathic epilepsy. 8 were female and 12 were male. Prevalence of the epilepsy was 7.44% before the modification of the mating system (2005–2012; *N* = 121) and 3.90% in the post-management period (2013–2019; *N* = 282), although the difference is not statistically significant (Fisher’s exact test, *p* = 0.140). Qualified keepers reported normal development for all the affected cubs. All the cubs were considered normal in the interictal period with an unremarkable physical and neurological examination. The age at ES onset was less than 6 months for 19 cubs and 26 months for the remaining cub (considered adult-onset; case 12). The average age at ES onset was 75 days (60–157) for the 19 cubs younger than 6 months at ES onset. A total of 647 ES was recorded and the mean number of ES per animal was 32 (3–248). Incidence of ES varied between isolated to cluster ES. All the cubs presented cluster ES. No prodromal signs were identified. The ictal phase ranged from focal ES, focal progressing to generalized ES and generalized ES; the mean duration was 1 min, 53 s (3 s- 5 min). ES did not occur during a specific time of the day. Epileptic seizure triggers were not identified however, in 1 cub ES seemed to coincide with arousal during playing periods.

Focal ES included hypersalivation, facial twitching affecting the ears or eyelids, unilateral eye blinking, nose-licking, repeated jerking of the head and oroalimentary automatism (mainly chewing and swallowing). Most of the cubs were sitting or lying laterally at this stage. Paroxysmal episodes of anxiousness, restlessness, fear reactions, disorientation, seeking and staring episodes that could not be easily interrupted by demanding attention were additionally observed. These suspected behavioural ES occurred occasionally combined with motor activity of the head, neck and mainly the forelimbs. The described clinical signs were occasionally followed by generalized tonic-clonic ES. At least in one cub, additional clinical signs that either stopped or progressed with three different stereotypical patterns of progression were detected. One pattern showed an increased intensity of the previous focal signs, progressive extension of the neck and loss of balance. In the second pattern, after loss of balance, the cub initiated walking backwards followed either by sudden recovery or followed by falling sideways or backwards (sometimes repeated cycles). The third pattern consisted of progression of the signs described in the second pattern to a generalized tonic-clonic ES. During the post-ictal phase, disorientation, staring, ataxia, compulsive locomotion, restlessness, tiredness and blindness were sporadically recorded. Postictal myoclonus involving the head, neck, and forelimbs musculature were recorded in one case. In this patient, postictal myoclonus lasted from some hours to several days after generalized ES (case 20).

### Diagnostic investigations

None of the cubs had significant abnormalities on minimum data base blood tests, infectious diseases testing, and urinalysis. Brain MRI and CSF analysis results performed in 3 lynx cubs were unremarkable. Although reference values for Iberian lynx CSF were not previously reported, references for dogs and cats were considered. Quality tests of the water, food (including live prey), enclosure and enamelware could not identify any underlying cause for ES. Consequently, underlying conditions provoking ES were ruled out and benign juvenile idiopathic epilepsy was strongly suspected.

### Treatment

All the cubs except one (case 4) received long term AED treatment. AEDs used included oral administration of Phenobarbital, Imepitoin, Levetiracetam, Pregabalin and Zonisamide. Phenobarbital was elected as a first line drug in 19 cubs. The dose was variable due to altering body weights as the cubs developed. An initial dose of 1–2 mg/kg every 12 h was started and progressively increased as needed. Phenobarbital highest dose used for the cubs was 5 mg/kg every 12 h. Side effects were mainly observed during the first 2 weeks of treatment or during a few days after a dose increase. These signs included somnolence, ataxia, weakness, reduced activity and mild increase of liver enzymes. Phenobarbital serum concentration was monitored 1 month after starting the treatment. Given the wild nature of the patients, blood tests were performed under general anaesthesia thus, serum concentration monitoring was not systematically performed every 3 months. If the cubs were anaesthetised for other procedures, rechecks would be performed at these time points. Steady-state phenobarbital serum concentration was within therapeutic range reported for cats (12.7–45.4 μg/ml) [10] and dogs (15–40 μg/ml) [11] at all measurements. One cub receiving 5 mg/kg every 12 h had a raised value of 59.9 μg/ml at 6 weeks after starting the treatment but showed no side effects of the medication at any time.

Polytherapy was necessary in seven lynxes (35%), Table 1. Imepitoin was used as an additional medication in 6 lynxes, either to increase ES control or to allow faster discontinuation of phenobarbital for one cub. Imepitoin was introduced on an initial dose of 10 mg/kg every 12 h and progressively increased, as needed (maximum dose 40 mg/kg). The mean duration for this treatment was 1007 days (182–1797). Mild sedation was observed especially in the first days of treatment. Levetiracetam, Pregabalin, and Zonisamide were progressively used only in 1 lynx that was considered pharmacoresistant (case 20). The initial dose for levetiracetam was 12.5 mg/kg every 12 h and it was progressively increased to 37 mg/kg/ every 12 h. The length of the treatment was 554 days. Pregabalin was used on a dose of 2.5 mg/kg every 12 h. Zonisamide was commenced on a dose of 10 mg/kg every 24 h and then increased to 20 mg/kg every 24 h. No significant side effects were reported for the additional drugs apart from mild to moderate sedation. The duration of Pregabalin and Zonisamide treatment was 8 and 4 months respectively.

**Table 1:** Summary of treatment and outcome

Case	Sex	ASO	ATO	ALRS	AET	Treatment	Outcome
**1**	M	75	78	104	896	PHB	Epilepsy remission. Died at 3920 days. Postmortem: CRD
**2**	M	71	73	107	610	PHB	Epilepsy remission. Phenobarbital dose 0.44 mg/kg/SID since day 373. Died at 631 days. Postmortem: CRD.
**3**	M	116	124	125	712	PHB	Epilepsy remission. Died at 1409 days. Postmortem: CRD
**4**	M	157	–	–	191	–	Died at 192 days. Postmortem: Bacterial septicaemia (*Salmonella spp*)
**5**	M	75	77	457	Unfinished	PHB	ES remission. Phenobarbital dose 0.6 mg/kg/SID since day 1537. Current age 3543 days
**6**	M	63	64	76	Unfinished	PHB	Died at 134 days. Postmortem: Oesophageal foreign body.
**7**	F	69	82	1599	Unfinished	PHB, IMP	Died at 2526 days. Postmortem: Cardiac insufficiency.
**8**	F	62	66	87	452	PHB	Epilepsy remission. Current age 2845 days.
**9**	F	77	78	77	482	PHB	Epilepsy remission. Current age 2835 days.
**10**	F	64	66	398	Unfinished	PHB	Died at 428 days during cluster ES. Postmortem: Multiorgan failure and DIC associated with haemorrage and oedema affecting piriform and temporal lobes bilaterally.
**11**	F	64	65	66	99	PHB	Epilepsy remission. Current age 2101 days.
**12**	M	795	796	857	Unfinished	PHB, IMP	ES remission. Current age 2104 days.
**13**	M	74	75	140	Unfinished	PHB	ES remission. Phenobarbital dose 0.46 mg/kg/SID since day 522. Current age 1738 days.
**14**	M	67	68	68	1648	PHB	Epilepsy remission. Phenobarbital dose 0.42 mg/kg/SID since day 522. Current age 1738 days.
**15**	F	63	72	827	Unfinished	PHB, IMP	Euthanasia performed at 853 days due to a severe deterioration and poor quality of live. Postmortem: no significant findings.
**16**	F	65	71	900	Unfinished	PHB, IMP	ES remission. Current age 1753 days.
**17**	M	66	70	317	Unfinished	PHB	ES remission. Current age 1398 days.
**18**	M	63	67	451	Unfinished	PHB, IMP	Died at 451 days. Postmortem: Bacterial septicaemia (*Streptococcus spp*)
**19**	M	60	80	79	361	PHB, IMP	Epilepsy remission. Imepitoin started due to an early withdrawal of phenobarbital. Current age 956 days.
**20**	F	73	75	1148	Unfinished	PHB, LVT, PGB, ZNS	Euthanasia performed at 1157 days due to a severe deterioration and poor quality of live. Postmortem: no significant findings.

Ages in days. *ASO* age at seizure onset, *ATO* age at treatment onset (phenobarbital), *ALRS* Age at last reported seizure, *AET* age at the end of treatment, *PHB* Phenobarbital, *IMP* imepitoin, *LVT* levetiracetam, *PGB* pregabalin, *ZNS* zonisamide, *CRD* chronic renal disease, *ES* Epileptic Seizure, *DIC* Disseminated intravascular coagulation

### Outcome

Eight out of twenty cubs (40%) were seizure free 10 days (0–34) after starting treatment. In these animals, treatment was discontinued after a mean of 578 days of no further ES (mean follow-up longer than 5 years). Epilepsy remission was considered for these cubs. Eleven cubs (55%) and the one considered adult-onset (5%), continued on AED treatment for a mean of 1306 days (70–3466). Of those, five lynxes (25%) were considered in ES remission and no further ES were reported for an average of 2 years. Consequently, 65% of the affected lynx cubs were seizure free for more than 6 months.

Mortality during the time of the study was 50% (10 lynxes). Chronic renal disease was the underlying cause of death in 3 out of 10 lynxes [1, 12] at the age of 1 year and 9 months (case 2), 3 years and 10 months (case 3), and 10 years 9 months (case 1) respectively. All of them belonged to the group which achieved epilepsy remission and AED treatment had been discontinued. Seven out of ten animals died during an ES or after severe ES. Underlying fatal condition could be identified in four of them. Cardiac insufficiency (case 7), bacterial septicaemia (*Streptococcus spp*) (case 18), bacterial septicaemia (*Salmonella spp*) (case 4), and oesophageal foreign body (case 6) were identified in the post-mortem examination. The age at death was 6 years and 11 months, 1 year and 3 months, 6 months, and 4.5 months respectively. One cub died during an episode of cluster ES at the age of 1 year and 2 months (case 10). Euthanasia was elected and performed due to a severe deterioration of general condition and poor quality of life at the age of 2 years and 4 months in one lynx (case 15), and 3 years and 2 months in another (case 20) Table 1.

For the ten surviving animals, treatment was discontinued in 5 cases, and no further ES have been reported after 7 years and 6 months (case 8), 7 years and 9 months (case 9), 5 years and 9 months (case 11), 4 years and 9 months (case 14), and 2 years and 7 months (case 19) respectively.

**Fig. 1:**
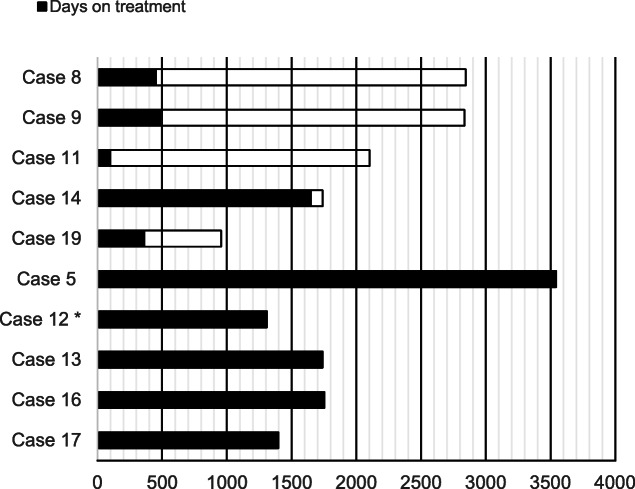
Bar plot for treatment duration and follow-up for lynxes that were still alive at the end of the study. The figure illustrates the relation between the duration of treatment (black bars), follow-up (white bars) and last reported seizure (vertical white mark). The last 5 cases have equal treatment duration and follow-up value

Three lynxes are still on phenobarbital treatment with no more reported ES after 2 years and 11 months (case 17), 4 years and 9 months (case 13), and 8 years 4 months (case 5) respectively. Two of these three animals were receiving a low dose of phenobarbital (0.46 and 0.6 mg /kg every 12 h) at the time of writing. Two more animals are still on polytherapy with no more ES reported after 2 years and 4 months (case 16) and 3 years and 7 months (case 12) of treatment respectively. Interestingly, the lynx on polytherapy that did not have more ES after 3 years and 7 months is the one considered a late onset (Fig. 1).

### Pedigree analysis

Genetic findings showed that the mean kinship between pairs of affected individuals was higher than that of pairs of non-affected individuals plus pairs of affected and non-affected individuals (*k*_aff_ = 0.128, SD = 0.0003; *F*_nonaff_ = 0.120, SD < 0.0001; t-test: t = − 2.641, *p* = 0.008). Affected individuals were on average more inbred (*F*_aff_ = 0.101, SD = 0.041; *F*_nonaff_ = 0.077, SD = 0.048; Kruskal-Wallis test: Z = 2.21, *p* = 0.027), had a larger proportion of genetic ancestry from Andújar (*θ*_*S*aff_ = 0.713, SD = 0.337; *θ*_*S*nonaff_ = 0.509, SD = 0.365; Kruskal-Wallis test: Z = 2.436, *p* = 0.015), and a lower hybridity than non-affected individual (*θ*_*H*aff_ = − 0.425, SD = 0.674; *θ*_*H*nonaff_ = − 0.059, SD = 0.697; Kruskal-Wallis test: Z = − 2.303, *p* = 0.021).

## Discussion

This retrospective study describes suspected benign juvenile idiopathic epilepsy in 20 out of 403 captive Iberian lynx cubs. This has been characterized by focal ES, focal ES progressing to generalized ES, and generalized ES without any underlying disorder, and seizure onset under the age of 6 months old. One of the cases had an adult onset. ES remission was 65%.

Idiopathic epilepsy has been well described in domestic animals and has also been mentioned in lynxes [1]. Epilepsy is defined as a disease of the brain characterized by an enduring predisposition to generate ES in a frequency of at least 2 unprovoked episodes more than 24 h apart [3]. Idiopathic epilepsy is considered a disease with the following categories: a causative gene is identified/confirmed (genetic epilepsy), a genetic suspicion is supported by a high prevalence in the breed (suspected genetic epilepsy) and epilepsy in which an underlying cause is suspected but it remains unknown without evidence of structural epilepsy (epilepsy of unknown cause) [3]. The presence of a normal interictal neurological status and the absence of a detectable underlying brain abnormality are the main diagnostic criteria to consider idiopathic epilepsy in animals and humans. The main differential diagnosis for focal ES are paroxysmal dyskinesias. Paroxysmal dyskinesias are an abnormal, sudden and involuntary contraction of a group of skeletal muscles and can be difficult to differentiate from focal ES [13]. The clinical presentation of our lynx cub population is suggestive of ES rather than dyskinesia. When ES first presented in a litter of lynxes, underlying causes such as infectious, toxic or metabolic conditions were considered, particularly as some of the affected cubs had been hand-reared and sudden severe cluster ES were an unusual presentation for epileptic patients. Additionally, giving the stringent rules of the breeding program about environment, feeding and management [14], the quality of the water, food (including live prey), enclosure and enamelware were also tested. Thorough investigations could not identify any underlying cause for the ES.

The international veterinary epilepsy task force consensus currently divides idiopathic epilepsy into three tiers of confidence, based on the level of investigations that are performed [15]. This is based upon findings in companion animals, however a parallel could be used with our study population. Following the guidelines from the consensus, the majority of lynx cubs could qualify for Tier I confidence level for the diagnosis of idiopathic epilepsy. Only 3 of them were classified as Tier II confidence level [15]. The hazard ratio for age at onset in idiopathic epileptic dogs has been established in 6 months to 6 years and it has been used as a diagnostic standard [15]. A younger age of onset (3 months) has been recently described in cats with epilepsy of unknown cause [16]. Martinez et al. [1] reported an early onset of idiopathic epilepsy in a morbidity and mortality study in captive Iberian lynx. Furthermore, benign epileptic syndromes affecting young patients has been described in humans (childhood epilepsy) [4, 17–19], in Lagotto Romagnolo dog [5], Egyptian Arabian foals [6] and recently in one lynx cub [7]. One main characteristic shared between these benign forms of epilepsy, is that ES mainly occur in childhood /adolescence and cease in adulthood [4, 18–20], as observed in the here presented lynx population. Benign idiopathic epilepsy in veterinary medicine is a rare condition. Jokinen et al. (2007) described benign familial juvenile epilepsy in 25 Lagotto Romagnolo puppies with both, isolated and cluster focal or secondary generalized ES [5]. The age at ES onset ranged from 5 to 9 weeks and ceased at mean age of 10 weeks (range 7.5 to 13 weeks). Puppies with most severe ES also had signs of neurologic disease including generalized ataxia and hypermetria. An adult-onset was reported for 3 dogs. Only 2 out of 25 animals received phenobarbital. One received two courses of AED treatment from the age of 7 to 11 weeks and after ES relapse at the age of 13 weeks. One month after ES relapse, phenobarbital was gradually discontinued. The other dog experienced cluster ES at the age of 2 years and 1 month, was started on phenobarbital and remained on medication in the long term. Spontaneous remission was reported for the survival puppies. In our study, all the cubs presented cluster ES thus, AED treatment was necessary, and it was continued until the ES went into remission. It is important to remark that the first 3 affected cubs had the longest period of treatment (longer than 18 months) before starting AED withdrawal, as this condition was new in the breeding program and there was a concern about treatment interruption. ES remission was 65%, however, epilepsy remission could only be considered in 40% of the cubs, as 25% of them are still on treatment. A causative mutation has been identified in the LGI2 gen for the *Lagotto romagnolo* canine breed [21]. However, the presence of clinical signs in the interictal period and structural findings affecting the cerebellum are more consistent with a neurological syndrome, rather than a form of epilepsy as it has been considered in our lynx population. Aleman et al. (2006) described juvenile idiopathic epilepsy in 22 Arabian foals [6]. ES were characterized by generalized tonic and clonic motor activity, staring, and loss of consciousness. Favourable response to phenobarbital in addition to potassium bromide in 3 of 22 foals was reported. Medication was progressively removed after 6 months of treatment and ES cessation occurred by one year. Both studies (Jokinen & Aleman) revealed interictal electroencephalographic findings consistent with epileptiform activity, which could not be assessed in our study. Minguez et al. (2019) described a case report of a lynx affected of suspected benign juvenile focal epilepsy. The same pattern of epilepsy was followed by most of the cubs in this study [7]. An adult-onset was observed for one lynx (5%); and several animals, as reported in dogs, required AED treatment in adulthood.

In humans, childhood-onset epilepsy syndromes range from moderately common and more benign disorders to epileptic encephalopathies with a variable prognosis [18–20]. Benign epilepsies during infancy are a wide group of epilepsies and comprise three identifiable electroclinical syndromes recognized by the International League against Epilepsy (ILAE): Benign Childhood Epilepsy with Centrotemporal Spikes (BCECTS) also called Benign Rolandic Epilepsy, Panayiotopoulos Syndrome (PS) and Idiopathic Childhood Occipital Epilepsy of Gastaut (ICOE-G) [4, 19]. BCECTS and ICOE-G are two of the common benign disorders which have several similarities to the juvenile form of epilepsy described in veterinary medicine. Benign partial epilepsy of infancy/Benign Familial Infantile Epilepsy is another juvenile epileptic syndrome with an infant onset [22, 23]. The affected children present with clusters of ES characterized by decreased responsiveness, staring, mostly with automatisms, and mild convulsive movements. Apnoea, cyanosis, and multifocal convulsions can also occur, and ES onset is usually in an age below 24 months. A single AED is usually sufficient, carrying an excellent prognosis for ultimate ES remission [10, 20, 23]. These clinical features and the progression of the disease are comparable to the here presented lynx population. The most similar syndrome to the presented cases would be Rolandic Epilepsy, which has as common cardinal features the presence of hypersalivation, unilateral facial sensory-motor ES that may spread to the ipsilateral hand and oro-pharyngo-laryngeal symptoms. Consciousness is fully retained in more than half of the children with Rolandic ES and in the remainder, consciousness becomes impaired during the ictal period and progresses to hemiconvulsions or generalized tonic–clonic ES [4, 20]. This pattern was also observed in our cubs. The total number of ES is low, the majority of patients having fewer than 10 ES with 10–20% having just a single ES [4]. In the present study, the mean number of ES, excluding the lynx considered drug-resistant, was 21 (range 3–57). ES disappeared regardless of previous drug resistance to treatment, which may be present in up to 20% of patients [20]. In our study, drug resistance was suspected in animals that had to receive polytherapy (35% of patients), and it was confirmed in one lynx that was finally euthanatized due to a poor quality of life. This patient presented with postictal myoclonus lasting from some hours to several days after generalized ES, which can resemble opercular status epilepticus that can occur in children with atypical evolution of Rolandic Epilepsy [4]. Opercular status epilepticus consists of ongoing unilateral or bilateral contractions of the mouth, tongue or eyelids, perioral or other myoclonus, difficulties in swallowing, buccofacial apraxia, and hypersalivation lasting for hours to months [4]. The risk of developing generalized tonic-clonic ES in adult life in Rolandic Epilepsy is less than 2% [20]. It is suspected that the benign childhood focal ES syndromes are likely linked by a genetically determined, functional derangement of the systemic brain maturation also called “benign childhood seizure susceptibility syndrome” that is mild and age-related [4]. It is unclear if this also exists in veterinary patients, although it is suspected given the similarity in the clinical presentation in multiple species [5–7, 24].

Ten out of twenty lynxes died during the time of the study (mortality 50%). Different underlying fatal conditions were detected in at least 5 lynxes, including chronic renal disease, cardiac insufficiency, bacterial septicaemia (*Streptococcus spp,* and *Salmonella spp*), and oesophageal foreign body. The cub with isolated Salmonella spp. had not received long-term seizure treatment. This cub initially displayed 25 focal ES in 24 h, and ES were controlled after several diazepam boluses. The cub recovered within 4 h after the last ES. A potential intoxication due to aerial fumigation with diflubenzuron 1.5% was first suspected but it could not be confirmed. The cub stayed neurologically normal during the following weeks however, thirty-five days after recovering, respiratory and digestive signs were acutely observed. ES relapsed, and the cub died after 2 more ES. The histopathology examination revealed hyperacute necrosis of the Central Nervous System secondary to the systemic disease. ES were suspected to be a consequence of the systemic process; however, ES relapse in the natural course of epilepsy could not be ruled out.

Although spurious or indirect effects cannot be discarded, the higher average kinship between epileptic lynx than between healthy individuals indicates a genetic component of the disease. This is in line with suggestions of a causative genetic basis for idiopathic epilepsy in humans and domestic animals, including the domestic cat [24]. A lower hybridity and higher inbreeding of affected individuals suggest a (partial) recessive mode of inheritance and add evidences to previous evidences of inbreeding depression and high genetic load in Iberian lynx. Finally, the higher *S* of affected individuals suggests a higher frequency of the corresponding alleles in Andújar than in Doñana, what is consistent with their genetic isolation for about 200 years and their high level of genetic differentiation suggested by molecular markers and genome-wide data. The occurrence of two affected cases in first generation offspring of inter-populational crosses suggest however that the epilepsy allele(s) may be also present in Doñana or that deleterious alleles are not completely recessive. Assuming a recessive inheritance mode, 29 individuals (14 males and 15 females) which produced affected offspring (16 of which were founders; 7 males and 9 females) were identified as potential carriers. Mating between carriers have been avoided since 2012, whereas affected individuals were completely excluded from reproduction. This modification could have influenced the decrease in prevalence observed in the period 2012–2019 (3.9%), with no more cases being detected in 2018 and 2019.

The main limitations of this study are those associated with a retrospective investigation and wild and endangered nature of the animals. Cases were selected based on clinician diagnostic suspicion. Only 3 animals had an MRI scan and CSF analysis to rule out a structural cause for ES, equally none of the cubs had electroencephalography studies which would have been useful to characterise the epilepsy further. Considering the wild nature of the animals, further investigations and diagnostics posed a challenge. However, clear records kept for each animal and their assessment by a residency trained veterinary neurologist could provide sufficient support for our findings. Moreover, a very long follow-up (up to 10 years) without neurological signs apart from the ES, may provide significant evidence to rule out any underlying disease as a potential cause of the here presented epilepsy.

## Conclusion

The presented study reports the occurrence of idiopathic epilepsy affecting 20 Iberian lynxes of a captive breeding colony. The clinical features and suspected self-limited course are consistent with benign juvenile epilepsy reported either in humans or other animal species. The presented study provides evidence that juvenile benign form of idiopathic epilepsy occurs in the Iberian lynx. Information obtained from this study strongly suggests a genetic basis for the here presented epilepsy in this species, what may have a significant impact in the management of further breeding programs.

## Methods

### Animal population

A total of 533 lynxes were born in the Iberian lynx captive breeding program 2005–2019. Only 403 lynxes out of 533 survived longer than 60 days (the earliest age at ES onset) and were able to develop the here presented form of epilepsy. The animals in this breeding programme were founded from two genetically differentiated populations in Andalucía, one near Andújar-Cardeña in Sierra Morena and the other in the Doñana region [14]. In 2012, given the suspicion of a genetic basis of this epilepsy, mating between presumed carriers was avoided. Medical records from the breeding program were reviewed and cubs initially selected if they presented with more than one unprovoked ES within a 6-month period. Data taken from the records included information for signalment, history, physical and neurological examinations, clinical signs including number of ES prior and after diagnosis and treatment, ES semiology, ES classification and time until suspected remission based on 24 h video recordings. Video footage of ES and episodic abnormal movements or behaviour were selected by the veterinary surgeons of the breeding centre for ultimate analysis by a ECVN resident trained neurologist. Due to handling difficulties, the majority of cubs were neurologically assessed via video analysis, however 4 did have a full neurological examination, all of them performed by the same ECVN resident trained neurologist.

### Diagnostic investigations

Diagnostic test performed included CBC, and serum biochemistry analysis (glucose, creatine kinase, lactate dehydrogenase, aspartate aminotransferase, alanine aminotransferase, alkaline phosphatase, gamma-glutamyl transferase, bile acids, sodium, chloride, potassium, calcium, magnesium, phosphate, iron, urea, creatinine, uric acid, total protein, albumin, globulins, cholesterol, triglycerides, amylase, lipase and protein electrophoresis) and urinalysis.

A full profile of infectious diseases was also performed. Canine Distemper virus, Feline Coronavirus, Feline Leukaemia virus, Feline Immunodeficiency virus, Feline Parvovirus, Feline Herpesvirus-1, Feline Calicivirus, *Anaplasma phagocytophilum*, Anaplasma spp., Babesia, *Bartonella hensalae,* Brucella, *Candidatus Mycoplasma [CM] turicensis,* CM *haemominutum*, *Clamydophila felis*, *Cytauxzoon felis*, and Toxoplasma gondii were ruled out by PCR and serology test from blood samples. For viral infectious diseases additional oropharyngeal, conjunctival and faecal PCR test were performed. Furthermore, microbiological analysis and the presence of heavy metals and insecticides in the water, food, enclosure and enamelware were also tested.

Magnetic resonance imaging (MRI) of the brain was performed in 3 cubs using a 1.5 T unit (Sigma Infinity Echospeed Plus, General Electric) in one patient and a 0.2 T unit (ESAOTE Vet MRI) in the remaining two. Acquired sequences included 3 planes T2-weighted, dorsal (1.5 T MRI unit) and transverse (0.2 T MRI unit) fluid-attenuated inversion recovery (FLAIR) sequence, and dorsal and transverse T1-weighted images pre- and post-contrast administration (gadoteric acid 0.5 mmol/ml, Dotarem-Guerbet). Imaging studies were performed under general anaesthesia. The cubs were premedicated with an intramuscular injection of dexmedetomidine 10 mcg/kg, (Dexdomitor 0.5 mg/ml, Zoetis), methadone 0.4 mg / kg (Metasedine 10 mg/ml, Esteve) and midazolam 0.2 mg / kg (Midazolam Normon 5 mg/ml). General anaesthesia was induced with intravenous propofol 1 mg/kg (Vetofol 1%, Esteve) and maintained with Isofluorane (Isoflo, Esteve) and oxygen. Cerebrospinal fluid (CSF) was obtained from the cerebellomedullaris cistern after the MRI studies. CSF analysis encompassed total protein (TP), total nucleated cells count (TNCC) and cytology.

### Treatments

All the cubs received early symptomatic treatment with AED. Data on AED doses, blood AED levels monitoring, treatment duration, suspected remission and relapses were recorded. Given the nature of the breeding program, long term follow-up up to ten years was possible. All the affected lynxes were treated according to medical regulations and animal care.

### Outcome

No ES for more than 6 months was considered a potential epilepsy remission. AED treatment was then tapered monthly by 25% over 4 months and finally discontinued. Long term follow-up was included up to ten years after initial diagnosis. All animals were maintained in the breeding program or in Zoo Exhibition (including those that could not discontinue the AED treatment). As an endangered species, euthanasia required special consideration and was considered only if there was significant concern of a poor quality of life or a bad prognosis. Any lynxes euthanised or that died to different causes were included in the follow-up data. If euthanasia was performed it was done so under previous anaesthesia using an intramuscular injection of either tiletamine hydrochloride plus zolazepam (10 mg/kg body-weight; Zoletil; Virbac, Carros, France) or a combination of ketamine hydrochloride (5 mg/kg; Imalgene 1000; Merial,Lyon, France) plus medetomidine hydrochloride (50 μg/kg;m Domtor; Orion Pharma, Espoo, Finland). After being anaesthetized, euthanasia was performed by intravenous administration of pentobarbital (100 mg/kg; Alfasan Nederland BV, Woerden, Netherlands) in one case, and by intravenous administration of T-61 solution (1 ml/kg; Tanax solution containing per ml: 200 mg embutramide, 50 mg mebezonium, 5 mg tetracaine; MSD Animal Health, Salamanca, Spain) in the other case. Post-mortem examination was performed by a qualified pathologist at Centro de Análisis y Diagnóstico de la Fauna Silvestre (CAD).

### Pedigree analysis

The contribution of genetics to the susceptibility to the disease was assessed by analysing the differences in ancestry and inbreeding coefficient between affected and non-affected individuals, and by testing for differences in average kinship between pairs of affected individuals versus the rest of possible pairs. Ancestry was captured by the indices *S* (source), calculated as the expected proportion of the genome coming from the Andújar genetic stock, and hybridity (*H*), or the expected proportion of loci in genome with one allele from Doñana and one from Andújar. Both indices were transformed into *θ*_*S*_ (*θ*_*S*_ = 2*S*-1) and *θ*_*H*_ (*θ*_*H*_ = 2*H*-1) [25]. Individual inbreeding and pairwise kinship were estimated from the genealogy of the captive population taking into account the relatedness among founders, which was estimated from microsatellite genotypes [26].

## Data Availability

The raw data supporting the conclusions of this article is available from the above-mentioned breeding centres on reasonable request.

## Abbreviations

AED: Antiepileptic drug
ES: Epileptic seizures
CBC: Complete blood count
MRI: Magnetic resonance imaging
CSF: Cerebrospinal fluid
TP: Total protein
TNCC: Total nucleated cell count
AED: Antiepileptic drug
S: Source
H: Hybridity
ILAE: International League against Epilepsy
BCECTS: Benign Childhood Epilepsy with Centrotemporal Spikes
PS: Panayiotopoulos Syndrome
ICOE-G: Idiopathic Childhood Occipital Epilepsy of Gastaut
